# Disproportionate Expression of ATM in Cerebellar Cortex During Human Neurodevelopment

**DOI:** 10.1007/s12311-023-01560-2

**Published:** 2023-04-29

**Authors:** Simon Deacon, William Dalleywater, Charles Peat, Simon M. L. Paine, Rob A. Dineen

**Affiliations:** 1 Department of Cellular Pathology, Nottingham University Hospitals NHS Trust, Nottingham, UK; 2 Faculty of Medical Sciences, Newcastle University, Newcastle, UK; 3 Department of Neuropathology, Nottingham University Hospitals NHS Trust, Nottingham, UK; 4 Mental Health and Clinical Neuroscience, School of Medicine, University of Nottingham, Nottingham, UK; 5 NIHR Nottingham Biomedical Research Centre, Nottingham, UK

**Keywords:** Cerebellum, Ataxia telangiectasia, Gene ontology, Transcriptome

## Abstract

Cerebellar neurodegeneration is a classical feature of ataxia telangiectasia (A-T), an autosomal recessive condition caused by loss-of-function mutation of the *ATM* gene, a gene with multiple regulatory functions. The increased vulnerability of cerebellar neurones to degeneration compared to cerebral neuronal populations in individuals with ataxia telangiectasia implies a specific importance of intact *ATM* function in the cerebellum. We hypothesised that there would be elevated transcription of *ATM* in the cerebellar cortex relative to *ATM* expression in other grey matter regions during neurodevelopment in individuals without A-T. Using *ATM* transcription data from the BrainSpan Atlas of the Developing Human Brain, we demonstrate a rapid increase in cerebellar *ATM* expression relative to expression in other brain regions during gestation and remaining elevated during early childhood, a period corresponding to the emergence of cerebellar neurodegeneration in ataxia telangiectasia patients. We then used gene ontology analysis to identify the biological processes represented in the genes correlated with cerebellar *ATM* expression. This analysis demonstrated that multiple processes are associated with expression of *ATM* in the cerebellum, including cellular respiration, mitochondrial function, histone methylation, and cell-cycle regulation, alongside its canonical role in DNA double-strand break repair. Thus, the enhanced expression of *ATM* in the cerebellum during early development may be related to the specific energetic demands of the cerebellum and its role as a regulator of these processes.

**Supplementary Information:**

The online version contains supplementary material available at 10.1007/s12311-023-01560-2.

## Introduction

Ataxia telangiectasia (A-T) is an autosomal recessive condition associated with cutaneous telangiectasias, cerebellar neurodegeneration, immunodeficiency, respiratory disease, and increased risk of cancer [1]. Defects in the ataxia telangiectasia mutated (*ATM*) gene lead to absent or defective production of the ATM gene product, a protein kinase with known roles in repair of double stranded DNA breaks and in regulating the cellular response to oxidative stress [2–4]. The ATM protein resides in an inactive homodimeric form and is recruited to DNA double-strand breaks via the Mre11-Rad50-Nbs1 (MRN) complex [5, 6]. By contrast, reactive oxygen species (ROS) trigger *ATM* activation in an MRN-independent manner, via a pathway dependent on the formation of a reversible disulphide bond between the two ATM monomers forming an active dimer [3, 7].

Patients with classical A-T have a complete absence of ATM protein or defective ATM protein with no detectable ATM kinase activity [8]. A-T patients who have defective ATM protein production with limited kinase activity are said to have variant A-T, and typically have a milder clinical phenotype. The neurological features range on this spectrum of severity and include progressive movement disorders, dystonia, oculomotor abnormalities, and classical cerebellar ataxia [8]. Pathologically, A-T is characterised by gross cerebellar atrophy involving both the vermis and hemispheres, with loss of Purkinje cells, thinning of the molecular layer, and depletion of granular neurones [9]. Macroscopically, the cerebellar hemispheres and vermis are atrophic [10],in classic A-T, the cerebellar atrophy progresses throughout childhood [11], whilst adults with variant A-T show a spectrum of cerebellar hemispheric and vermian atrophy ranging from none to severe [12]. In contrast to the prominent cerebellar neurodegeneration, atrophy of the cerebral grey matter (both cortical and deep) is not a feature of A-T, although quantitative imaging studies and systematic pathological studies are lacking.

Animal models have been extensively used to investigate the pathogenesis of A-T [13]. However, many of these models do not recapitulate the neurological features found in patients [14]. Recently, mouse models have been described using a double-hit method of dual null mutations in both the *ATM* and *APTX* genes in the same animal [15, 16]. APTX mutations cause the syndrome ataxia with oculomotor apraxia 1, which shares a similar cerebellar pathology to A-T. Importantly, this animal model does demonstrate a progressive ataxic phenotype associated with atrophy of the cerebellar molecular layer. These rodents demonstrate the pathological hallmarks of genotoxic damage within the Purkinje cells, including abundant R-loops, increased double-strand breaks and aberrant RNA splicing and gene expression [15]. Furthermore, damage-susceptible gene loci in the Purkinje cells of the double-mutant model demonstrated a markedly open chromatin architecture, increasing susceptibility to genotoxic damage [15]. The biophysical properties of Purkinje cells are significantly perturbed, altering their neuronal activity and causing a progressive reduction in spontaneous action potential frequency which correlates with both cerebellar atrophy and the ataxia phenotype [16]. These studies support the notion that genotoxic stress is a key driver of neurodegeneration in A-T and that Purkinje cells are particularly susceptible. However, it remains unclear why ATM-knockout models fail to display the ataxic phenotype of A-T, suggesting either that species-specific adaptations provide alternative pathways which reduce the cerebellar effects of ATM deficiency or that they fail to develop such features within their shortened lifespan [16].

Intriguingly, a recent study has assessed the correlation of cell-type expression profiles for target cerebellar disease genes between humans and mice [17]. Whilst the cell-type expression signatures of granule cells, oligodendrocytes, astrocytes, and Purkinje neurons were correlated between humans and mice, microglia were the least correlated. The human microglia-enriched genes *ATM, SETX*, and *VPS13D* had relatively reduced expression in murine microglia compared to other cell types. As such, the failure of A-T mouse models to recapitulate the human neurological phenotype may be related to the cell-type specific differential expression characteristics of microglia between humans and rodents [17].

Multiple putative molecular mechanisms contributing to neurodegeneration in A-T have been identified, including the accumulation of excess reactive oxygen species, aberrant protein aggregation and disrupted autophagy and mitophagy [18–20]. Although dysfunction of these molecular pathways due to ATM deficiency provides a possible explanation for neurodegeneration, it is not well understood why the burden of neurodegeneration in A-T is cerebellar, with relative sparing of the cerebral cortex and other cerebral grey matter areas. In a recent review, Shiloh has argued that Purkinje cells have a particular vulnerability to ATM deficiency that is multifactorial [21]. In addition to the susceptibility to oxidative DNA damage that all neurones are exposed to as non-dividing, long-living cells, Purkinje cells receive extensive excitatory synaptic inputs and have high firing rates with accompanying high metabolic demands and consequent elevated exposure to oxidative stress.

The increased vulnerability of Purkinje cells and other cerebellar neurones to degeneration compared to cerebral neuronal populations in individuals with A-T implies a specific importance of intact ATM function in maintaining health in these cerebellar neurones. Previous studies have shown that ATM is constitutively expressed throughout the cell cycle, suggesting that continuous transcription is required to maintain ATM activity [22, 23]. In post-mitotic neuronal tissue samples, ATM is predominantly cytoplasmic, suggesting a translocation of ATM during neuronal differentiation from its nuclear localisation in proliferating cells [24, 25]. ATM immunohistochemistry demonstrated that cerebellar neurons, and in particular Purkinje cells, were strongly immunoreactive during the late prenatal and early postnatal periods, subsequently followed by persistent moderate reactivity in Purkinje cells. Cerebellar granule cells were also immunoreactive during the gestational period, but, in contrast, the expression of the ATM protein markedly reduced by full-term. [25]. Pharmacological inhibition of ATM protein by KU55933 leads to upregulation of the ATM protein mediated at the transcriptional level, as demonstrated by a luciferase reporter assay of the promoter of ATM [26]. Furthermore, ionising radiation has been shown to lead to activation of the ATM promotor [27].

On this basis, we hypothesised that a specific importance of ATM in cerebellar neuronal health would be manifest as elevated transcription of *ATM* in the cerebellar cortex relative to *ATM* expression in other brain regions during neurodevelopment in individuals without A-T. To test this hypothesis, we analysed the pattern of region-specific *ATM* transcription across normal human development using the BrainSpan Atlas of the Developing Human Brain [28], comparing the *ATM* expression in the cerebellar cortex to *ATM* expression in other brain regions. In order to identify the biological processes potentially modulated by cerebellar *ATM* expression, gene-ontology over-representation analysis was performed on sets of genes correlated with *ATM* expression.

## Materials and Methods

### Dataset

To explore cerebellar cortical *ATM* expression profiles across normal human development, we accessed *ATM* transcription data from BrainSpan Atlas of the Developing Human Brain (©2010 Allen Institute for Brain Science. Available from: http://www.brainspan.org/) [28]. This dataset provides gene expression data in up to 16 specific brain regions using RNA sequencing and exon microarray techniques for up to sixteen targeted cortical and subcortical grey matter structures from 42 brain specimens from 8 weeks post conception to 40 years of age. The ‘*RNA-Seq Gencode v10 summarised to genes*’ dataset was downloaded on 16th January 2021 (www.brainspan.org/static/download.html). This data set contains RNA-Seq RPKM (reads per kilobase per million) values averaged to genes.

### Inclusion and Exclusion Criteria

For inclusion of specimens in this analysis, data for *ATM* expression from cerebellar cortical (or whole cerebellum in early fetal specimens) and at least 4 other non-cerebellar brain regions had to be available.

### Analysis of ATM Expression Across Time

Cerebellar cortical *ATM* expression was expressed as a *z*-score relative to average *ATM* expression in all other grey matter regions for each brain specimen. Significant elevation of cerebellar cortical *ATM* expression relative to average *ATM* expression in all other grey matter regions was defined as *z*-score > 1.96. We did not specify an a priori model for the shape of the curve for the predicted increase in *ATM* expression during neurodevelopment, but inspection of the data prompted post hoc analysis using Pearson correlation to assess the age trajectory of relative cerebellar cortical *ATM* expression in two phases: the early neurodevelopmental period (foetal and first year post-natal specimens), and post-natal neurodevelopment through to adulthood. Statistical significance of correlation was defined as *α* < 0.05.

To test whether any observed increase in relative cerebellar cortical expression of *ATM* during early development was reflective of increased relative cerebellar cortical expression of genes more generally during this period, we calculated *z*-scores for cerebellar cortical expression of all other genes included in the dataset, relative to expression of those genes in all other grey matter regions. Genes for which no expression was recorded in any brain region in any brain specimen were excluded from the analysis. The trend of cerebellar cortical gene expression across the age range of the specimens was examined by plotting the distribution of *z*-scores for all genes for each specimen. The *z*-score for cerebellar expression of *ATM* was then compared to the distribution of *z*-scores for all genes for each specimen.

### Pearson Correlation Analysis

In order to investigate the downstream networks modulated by cerebellar *ATM* expression, Pearson correlation of *z*-scores was used to assess the relationship between relative cerebellar *ATM* expression and relative cerebellar expression of all other genes. Correlational analysis was performed across the whole cohort. Genes with an average expression across all samples within the lowest quartile of the dataset were excluded from the analysis. Genes with less than 25 cerebellar *z*-scores, due to either missing data or lack of cerebellar sampling, were excluded from the analysis. Statistical significance of correlation was defined as *α* < 0.05. Significant positive correlation with ATM expression was defined as *r* > 0.6 and negative correlation with ATM as *r* <  − 0.6. Sets were then generated using these thresholds to define positively and negatively correlated gene sets, respectively.

### Gene Ontology Analysis of Genes Related to ATM

Gene overrepresentation analysis was performed independently on both sets using an online tool (http://pantherdb.org/webservices/go/overrep.jsp). Benjamin-Hochberg correction was performed to adjust statistical significance for multiple comparisons (corrected FDR values are included within the results). Graphical analysis was performed using the ClueGO application for Cytoscape [29] to illustrate significantly associated (*p* < 0.05) biological processes.

## Results

Cerebellar and multi-region non-cerebellar *ATM* transcription data was available for 30 of the 42 specimens from the BrainSpan Atlas of the Developing Human Brain, ranging from the 12th post-conception week to 40 years (supplementary materials S1). For 3 early-stage foetal specimens, *ATM* expression data for whole cerebellar samples was provided, but for all other specimens, the cerebellar cortical *ATM* expression was provided. Twelve of the 42 specimens were excluded due to lack of cerebellar or multi-region non-cerebellar *ATM* expression data (supplementary materials S2).

**Fig. 1:**
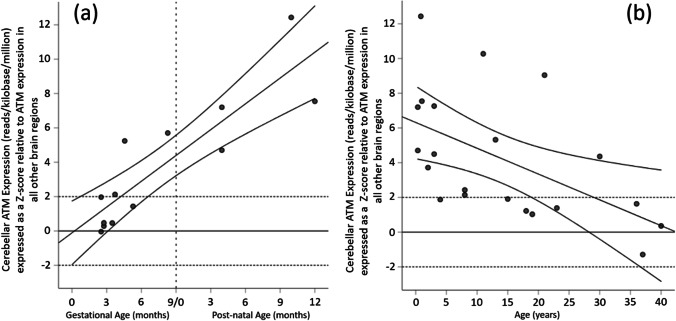
Scatterplot with linear fit line and 95% confidence intervals for cerebellar *ATM* expression (reads per kilobase per million) expressed as a *z*-score relative to *ATM* expression in all other brain regions plotted against age for the 30 included specimens from the BrainSpan Atlas of the Developing Human Brain (© 2010 Allen Institute for Brain Science). a Specimens from foetuses and infants during the first post-natal year, with the vertical dashed line indicating the end of the gestational period; and b post-natal specimens up to 40-years. The horizontal lines indicate z-scores of zero (solid line) and ± 1.96 (dashed line)

There was a steep linear increase in cerebellar cortical *ATM* expression relative to the average *ATM* expression in all other grey matter regions over the gestational period and first post-natal year (*r* = 0.87, *p* < 0.0005; Fig. 1a). Relative cerebellar cortical *ATM* expression was significantly elevated (*z*-score > 1.96) from mid-gestation onwards. There was a linear decline in cerebellar cortical *ATM* expression relative to average *ATM* expression in all other grey matter regions from infancy through to adulthood (*r* = -0.54, *p* = 0.011, Fig. 1b), although expression remained elevated (*z*-score > 1.96) during early childhood.

**Fig. 2:**
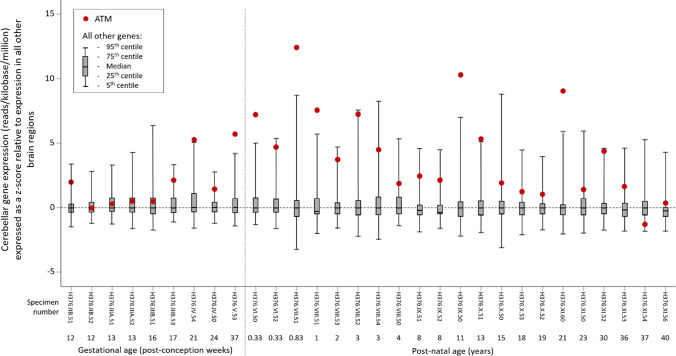
Boxplots showing the distribution of relative cerebellar cortical gene expression values (expressed as *z*-score relative to all other brain regions) for each brain specimen, with specimens arranged in ascending age order. Grey boxes indicate the interquartile range (with median shown), and whiskers represent the 5th and 95th centiles. Red dots indicate the *z*-score for cerebellar cortical *ATM* expression for each brain specimen. Vertical dotted line indicates the transition from the gestational to post-natal brain specimens

Of the 52,375 non-*ATM* genes reported in the dataset, 1899 genes were excluded from the analysis of the trend of cerebellar cortical gene expression due to the fact that these genes were not expressed in any brain region in any specimen (supplementary materials S3), resulting in 50,476 genes being included in this analysis (supplementary materials S4). There was no trend in increased relative cerebellar gene expression compared to expression in all other brain regions over the age range of the specimens (Fig. 2), with median values for the *z*-scores sitting close to zero for all specimens. Relative expression of cerebellar cortical *ATM* (compared to all other brain regions) was above the 95th centile for relative cerebellar cortical expression of all other genes (compared to all other brain regions) for 5 out of 7 specimens between 24 weeks post-conception to 1-year post-natal age, and for most specimens from early gestation onwards was above the 75th centile.

For the gene ontology analysis, genes within the lowest quartile of expression scores averaged across all samples (< 0.008109 RPKM) were excluded from the analysis as they provide little evidence for differential expression between the samples and reduce the likelihood of detecting significant results when correcting for multiple testing. Of the remaining 37,860 genes, all genes with greater than 5 missing *z*-score values within the 30 included specimens were excluded from this analysis. The total number of genes which were included in the gene ontology analysis was 31076 (supplementary materials S5).

**Fig. 3:**
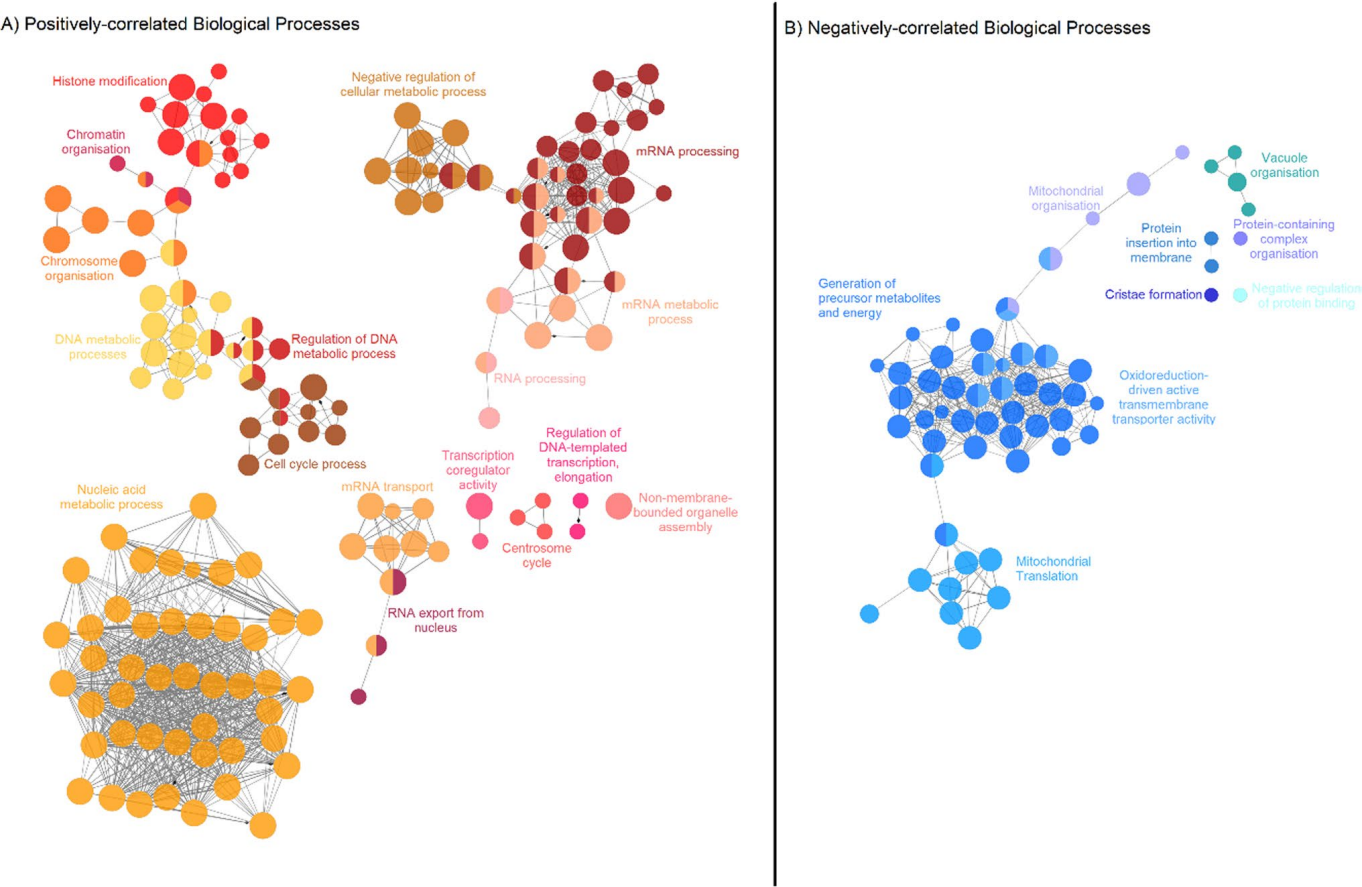
Network of gene ontology biological processes which are implicated in the set of genes correlated with cerebellar gene expression. Groups of related biological processes are clustered together and colour coded. The size of node represents *p*-value (*p* < 0.05). Networks were generated using the ClueGO plugin for Cytoscape [29]. The specific gene ontology biological processes represented by the nodes are demonstrated in the appendix (supplementary materials S7)

Gene ontology analysis demonstrated that differential networks of biological functions were significantly overrepresented in the positively and negatively correlated gene sets. In the set of genes positively correlated with cerebellar *ATM* expression (Fig. 3a) (supplementary materials S6), genes implicated in the canonical DNA double-strand break repair were over-represented (GO:0006302; fold change = 2.51, FDR = 0.002). Other biological processes over-represented in this set were regulation of biosynthetic and metabolic processes (GO:0031323, fold change = 1.67, FDR < 0001), regulation of cell cycle processes (GO:0010564 fold change = 1.72, FDR < 0.004), and histone modification (GO:0016570; fold change = 2.53, FDR < 0.001). RNA processing was also over-represented in the co-expressed set (GO:0006396; fold change = 2.34, FDR < 0.001), including RNA splicing (GO:0008380; fold change 2.84, FDR < 0.001) and mRNA metabolic processing (GO:0016071), fold change 2.67, *p* < 0.001). Biological processes under-represented in this set included aerobic respiration (GO:0009060, fold change = 0.01, FDR = 0.03) and generation of precursor metabolites and energy (GO:0006091, fold change = 0.26, FDR < 0.01).

The set of genes negatively correlated with cerebellar *ATM* expression included over-represented genes implicated in the related biological processes of mitochondrial organisation (GO:0007005, fold change = 2.98, FDR < 0.0001), mitochondrial electron transport (GO:0006123, fold change = 10, FDR < 0.01) (Fig. 3b), and oxidative phosphorylation (GO:0006119, fold change = 5.9, FDR < 0.0001). Aerobic respiration (GO:0009060, fold change = 4.99, FDR < 0.0001) and generation of precursor metabolites and energy (GO:0006091, fold change = 3.05, FDR < 0.0001) were also over-represented in this set, consistent with their under-representation in the set of genes positively correlated with cerebellar *ATM* expression, as above.

## Discussion

We identify a marked increase in cerebellar cortical *ATM* expression relative to expression in other brain regions during gestation and up to the end of the first year of life. Cerebellar cellular proliferation peaks approximately in the third trimester, followed by a gradual decrease that lasts up to the eighth postnatal month in the external granular layer, and a much more rapid decline in the internal granular layer [30]. We also observed a slow decline in expression from infancy onwards, with relative cerebellar cortical *ATM* expression generally remaining elevated during the early post-natal developmental period. The observed elevation in relative cerebellar cortical *ATM* expression does not appear to be due to a general increase in relative cerebellar cortical gene expression during this time, in that no overall increase was observed in the cerebellar cortical expression of > 50,000 other genes (compared to their expression in all other brain regions) over this developmental period. Indeed, in the late gestational and early postnatal brain specimens, relative expression of cerebellar cortical *ATM* was above the 95th centile for relative cerebellar gene expression.

This relative increase in cerebellar cortical *ATM* transcription implies a key role for *ATM* in healthy cerebellar development, and this may in part explain the disproportionate damage to the cerebellar cortex seen in *ATM* deficient individuals with A-T. The period of elevated relative *ATM* expression in these non-A-T specimens coincides with the characteristic and progressive childhood cerebellar atrophy seen in individuals with classic A-T. However, the mechanisms underlying the disproportionate effect of *ATM* loss of function on the cerebellum are still poorly understood [14, 21] and most *ATM*-knockout animal models, excepting the combined *ATM-* and *APTX-*knockout models, have failed to recapitulate the cerebellar phenotype observed in humans [19].

Our gene ontology analysis confirms the canonical function of *ATM* in double-strand break repair of DNA is implicated in cerebellar development, with expression of genes within this functional network, including MRE11, correlating with cerebellar expression of *ATM*. However, there is emerging evidence that, aside from the DNA damage repair function, *ATM* also mediates distinct pathways regulating redox homeostasis [3, 5, 31, 32]. Clinical studies in A-T patients have demonstrated deranged biomarkers of redox homeostasis [33, 34], supporting oxidative stress as an aetio-pathological mechanism in A-T. In keeping with this, our gene ontology analysis demonstrated that the cerebellar expression of genes implicated in the processes of aerobic respiration and oxidative phosphorylation, are negatively correlated with cerebellar expression of *ATM* throughout development.

The unique functional features of the cerebellum predispose an environment particularly susceptible to disruption in redox homeostasis. A characteristic feature of cerebellar Purkinje cells is their high excitatory input and synaptic firing rate, in which ATP plays a crucial role [35]. This energetic demand establishes a cellular environment highly vulnerable to oxidative stress [36]. Furthermore, the high transcriptional activity of Purkinje cells leads to a relaxed chromatin state in which the DNA is more susceptible to ROS-mediated damage [21]. ATM has been shown to translocate from the nucleus to the cytoplasm upon neuronal differentiation [24], and this may represent differential functions of ATM in dividing cells and post-mitotic neurones [37]. The double-strand break repair function must occur within the nucleus, where ATM is recruited to the double-strand break by the MRN complex. In contrast, ATM localisation has been shown to be predominantly cytoplasmic in mature neurones, suggesting a possibly different functional role in these cells [19]. Cytoplasmic ATM has been shown to be activated by ROS [37, 38]. These studies support the notion that aberrant redox homeostasis is a key aetiological mechanism in A-T cerebellar neurodegeneration.

However, ATM has a synergistic function as a regulator of mitochondrial biosynthesis and mitophagy, potentially integrating both redox-sensing and mitochondrial-regulatory roles as a master regulator of energetic homeostasis [39]. Mitochondrial dysfunction has been implicated in a broad range of neurodevelopmental and neurodegenerative diseases [40], including A-T, Bloom syndrome, and Nijmegen breakage syndrome, potentially forming the common pathogenic mechanism in these diseases [33, 34]. Indeed, rodent A-T models demonstrating impaired mitochondrial function responded to NAD^+^ replenishment therapy with restored mitophagy, suggesting this mechanism as a potential therapeutic target [41, 42]. Our gene ontology analysis particularly implicated a set of genes involved in mitochondrial organisation and biosynthesis. Previous studies have shown aberrant mitochondrial morphology in ATM deficient cells [43, 44]. Fibroblasts derived from A-T patients demonstrated depletion of mitochondrial DNA [45] and A-T thymocytes were demonstrated to have impaired mitophagy, in turn resulting in oxidative stress and ATP depletion [46]. ATM has also been shown to mediate mitophagy via phosphorylation of CHK2 and p53, thereby inducing transcription of denitrosylase S nitrosoglutathione reductase (GSNOR) [47].

There is an emerging novel epigenetic regulatory function of ATM [20, 48, 49]. Our data corroborates the relationship between *ATM* expression and histone methylation, with a functional group of genes implicated in this process positively correlated with cerebellar *ATM* expression. This set of genes included EZH2, previously implicated in A-T pathogenesis [50]. This study demonstrated that EZH2 is a target of ATM, and ATM-mediated phosphorylation of EZH2 reduces stability of the protein. EZH2 is a core component of polycomb repressive complex 2 (PRC2), a complex which mediates epigenetic trimethylation of histone H3 on Lys27 (H3K27me3). Thus, in A-T, ATM loss causes increased PRC2 formation and an alternation in the distribution of H3K27me3 marks, consequently leading to cell cycle re-entry and cell death of ATM-deficient neurones [50].

Other studies have also demonstrated a role of ATM in regulation of the cell cycle and cellular senescence, closely interlinked with the role of ATM in sustaining mitochondrial function [51]. Loss of neuronal cell cycle regulation by ATM appears to be an important factor in A-T (Y. [52], with both cortical and cerebellar neurones from ATM deficient mice showing extensive cell cycle re-entry and degeneration [53]. Our gene ontology analysis demonstrates that cerebellar expression of genes involved in the regulation of the cell cycle were positively correlated with ATM. Studies have shown that ATM kinase is a major mediator of DNA damage-induced NF-κB-mediated cellular senescence and the senescence-associated secretory phenotype (SASP) [54]. ATM, activated by oxidative stress, negatively regulates mechanisms promoting cell senescence by facilitating the removal of macroH2A.1 from SASP genes [55, 56]. Thus, the A-T phenotype may lead to premature cell senescence due to loss of this inhibitory mechanism, with increased senescent cells a feature of aging and age-related pathologies [51, 57].

### Limitations

Our analysis provides evidence for an important role of *ATM* cerebellar expression levels within a specific time-window, during the period of early neonatal development. Furthermore, we identified the likely functional significance of this change in cerebellar *ATM* expression, via gene ontology functional enrichment analysis. Whilst the RNAseq dataset used in this present study can only provide data on transcript abundance, rather than protein activity, our gene ontology analysis demonstrated that expression of *ATM* correlates with the expression of other genes implicated in canonical *ATM*-related biological processes. This supports our hypothesis that increased *ATM* expression is functionally relevant and that *ATM* is involved in the transcriptional regulation of related biological functions. However, it must be noted that there are additional considerations, such as post-transcriptional and post-translational modifications, which are not captured by this approach, and which may impact ATM activity in vivo [58]. Future research could investigate cerebellar ATM activity at the protein level, in order to ascertain the relationship between *ATM* expression and protein activity. In vivo work using modifiable *ATM* expression, such as tet-on/tet-off systems and knock out models of ATM, would complement our gene ontology analysis by demonstrating the role of *ATM* expression in the transcriptional regulation of these functionally relevant biological processes.

We have calculated a *z*-score to identify genes specifically upregulated in the cerebellum, due to its centrality in the pathogenesis of A-T. However, our data relies on bulk RNA-sequencing, which does not provide information on the differential expression of *ATM* and related genes between cell types. As such, our analysis assumes that the function of *ATM* is the same between cell types and that the biological processes mediated by *ATM* in bulk sequencing data are of relevance to the pathogenesis of A-T. Recent single-cell sequencing has shed light on the cell-specificity of these changes, finding that *ATM* is most highly expressed in microglia throughout development in both the cerebellum and prefrontal cortex, with cerebellar expression of *ATM* relatively lower in Purkinje cells [17]. The authors also show an increased proportion of glial cells in the A-T cerebellum and that A-T microglia demonstrate a highly perturbed transcriptome, with a significant number of differentially expressed genes (DEGs) [17]. This is consistent with previous studies implicating glial cells in the pathogenesis of A-T neurodegeneration [59–61]. Many of the DEGs were cell-type specific, with microglia and Purkinje cells harbouring the most cell-type-specific DEGs. Of these, less than 25% were identified in pseudobulk data analysis, underscoring the limitation of bulk RNA-sequencing. Future work could compliment this single-cell data by using spatial approaches and immunohistochemistry to identify the spatial distribution of *ATM* expression within the developing and adult cerebellum.

Whilst our dataset consists only of data from individuals without ataxia telangiectasia, we hypothesise that our findings are nevertheless of relevance to understanding the pathogenesis of A-T. The temporal window in which we have identified increased cerebellar expression of *ATM* corresponds to that of the onset of cerebellar symptoms in classical A-T. Identification of differential gene expression during this developmental window would provide evidence of its functional relevance to A-T pathogenesis, requiring comparison between matched non-AT and A-T RNAseq datasets across developmental timepoints.

Whilst the dataset includes samples throughout the developmental spectrum, it does not have specimens matched between timepoints. As such, the comparison between ages may be biased by secondary variables such as co-morbid conditions. Utilising larger matched datasets, with more replicates per time-point, would enable elucidation of differential gene expression between stages throughout normal development. Our gene ontology analysis across the whole cohort assumes that the function of ATM remains the same across development. Further research utilising more samples is required in order to conduct gene ontology analysis at multiple time-points, and ascertain if the biological functions of *ATM* changes during neurodevelopment.

Although we hypothesised that relative cerebellar cortical ATM expression would be elevated during neurodevelopment, we did not specify an a priori model for the shape of the curve. Consequently, the correlation analysis dividing the data into two, early and later neurodevelopmental phases, was post hoc following inspection of the data, and should be considered exploratory, requiring further independent validation.

## Conclusion

We provide data which indicates a degree of temporal specificity in the role of ATM in normal cerebellar development. This indicates a likely window in which ATM function is of particular importance and which warrants further investigation. We demonstrate a marked increase in cerebellar cortical *ATM* expression relative to expression in other brain regions during gestation that is sustained during early childhood. This observed maximal elevation in relative cerebellar cortical *ATM* expression coincides with the onset of the characteristic and progressive childhood cerebellar neurodegeneration seen in individuals with classic A-T.

The aetiological mechanisms which contribute to the cerebellar neurodegeneration characteristic of ataxia telangiectasia remain poorly understood. Alongside the canonical DNA repair function of ATM, our gene ontology over-representation analysis demonstrates multiple other functional networks are correlated with cerebellar *ATM* expression across the lifespan. The biological processes implicated in this network include mitochondrial function and cellular respiration, histone methylation, and cell cycle regulation, consistent with the published roles of ATM in regulating redox homeostasis, mitochondrial biosynthesis, and cellular senescence. The enhanced expression of *ATM* in the cerebellum during early development may be related to the specific energetic demands of the cerebellum, and our data shows that *ATM* appears to be involved in the regulation of these processes.

Emerging single-cell RNA-sequencing data demonstrates an important role of microglia in the pathogenesis of A-T [17]. However, the authors acknowledge that there is activation of microglia in the A-T prefrontal cortex also, despite a lack of cortical atrophy. This suggests that cerebellar neurons have a particular susceptibility to activated microglia compared to other brain regions, and that there other cerebellar-specific mechanisms by which A-T neurodegeneration occurs. Future work must reconcile this single-cell data with the disproportionate cerebellar neurodegeneration seen in A-T, by further delineating the contribution of specific cell-types to the onset of neurodegeneration and exploring the interaction of glial cells and neurons in mediating this phenotype.

### Supplementary Information

Below is the link to the electronic supplementary material.

Supplementary file1 (CSV 172 KB)

Supplementary file2 (CSV 9 KB)

Supplementary file3 (XLSX 14524 KB)

Supplementary file4 (XLSX 1632 KB)

Supplementary file5 (CSV 296 KB)

Supplementary file6 (XLSX 34 KB)

Supplementary file7 (PNG 351 KB)

Supplementary file8 (PNG 125 KB)

## Data Availability

Transcription data from BrainSpan Atlas of the Developing Human Brain is publicly available from: http://www.brainspan.org/ (©2010 Allen Institute for Brain Science.) (RRID:SCR_008083).

## References

[1] Rothblum-Oviatt C, Wright J, Lefton-Greif MA, McGrath-Morrow SA, Crawford TO, Lederman HM (2016). Ataxia telangiectasia: a review. Orphanet J Rare Dis.

[2] Chen K, Albano A, Ho A, Keaney JF (2003). Activation of p53 by oxidative stress involves platelet-derived growth factor-β receptor-mediated ataxia telangiectasia mutated (ATM) kinase activation. J Biol Chem.

[3] Ditch S, Paull TT (2012). The ATM protein kinase and cellular redox signaling: beyond the DNA damage response. Trends Biochem Sci.

[4] Lavin MF (2008). Ataxia-telangiectasia: from a rare disorder to a paradigm for cell signalling and cancer. Nat Rev Mol Cell Biol.

[5] Shiloh Y, Ziv Y (2013). The ATM protein kinase: regulating the cellular response to genotoxic stress, and more. Nat Rev Mol Cell Biol.

[6] Stracker TH, Petrini JHJ (2011). The MRE11 complex: starting from the ends. Nat Rev Mol Cell Biol.

[7] Guo Z, Deshpande R, Paull TT (2010). ATM activation in the presence of oxidative stress. Cell Cycle.

[8] Jackson TJ, Chow G, Suri M, Byrd P, Taylor MR, Whitehouse WP (2016). Longitudinal analysis of the neurological features of ataxia-telangiectasia. Dev Med Child Neurol.

[9] Clark HB. Degenerative ataxic disorders. In Greenfield’s Neuropathology-Two Volume Set (pp. 823–840). CRC Press. 2018.

[10] Tavani F, Zimmerman RA, Berry GT, Sullivan K, Gatti R, Bingham P (2003). Ataxia-telangiectasia: the pattern of cerebellar atrophy on MRI. Neuroradiology.

[11] Dineen RA, Raschke F, McGlashan HL, Pszczolkowski S, Hack L, Cooper AD, Prasad M, Chow G, Whitehouse WP, Auer DP (2020). Multiparametric cerebellar imaging and clinical phenotype in childhood ataxia telangiectasia. NeuroImage: Clin.

[12] Schon K, van Os NJH, Oscroft N, Baxendale H, Scoffings D, Ray J, Suri M, Whitehouse WP, Mehta PR, Everett N (2019). Genotype, extrapyramidal features, and severity of variant ataxia-telangiectasia. Ann Neurol.

[13] Lavin MF (2013). The appropriateness of the mouse model for ataxia-telangiectasia: neurological defects but no neurodegeneration. DNA Repair.

[14] Biton S, Barzilai A, Shiloh Y (2008). The neurological phenotype of ataxia-telangiectasia: solving a persistent puzzle. DNA Repair.

[15] Don Kwak Y, Shaw TI, Downing SM, Tewari A, Jin H, Li Y, Dumitrache LC, Katyal S, Khodakhah K, Russell HR, McKinnon PJ. Chromatin architecture at susceptible gene loci in cerebellar Purkinje cells characterizes DNA damage-induced neurodegeneration. Sci Adv. 2021;7(51):eabg6363. 10.1126/sciadv.abg6363 PMC11323782 34910524

[16] Perez H, Abdallah MF, Chavira JI, Norris AS, Egeland MT, Vo KL, Buechsenschuetz CL, Sanghez V, Kim JL, Pind M, Nakamura K, Hicks GG, Gatti RA, Madrenas J, Iacovino M, McKinnon PJ, Mathews PJ. A novel, ataxic mouse model of ataxia telangiectasia caused by a clinically relevant nonsense mutation. Elife. 2021;10:e64695. 10.7554/eLife.64695 PMC8601662 34723800

[17] Lai J, Kim J, Jeffries AM, Tolles A, Chittenden TW, Buckley PG, Yu TW, Lodato MA, Lee A (2021). Single-nucleus transcriptomic analyses reveal microglial activation underlying cerebellar degeneration in Ataxia Telangiectasia. BioRxiv.

[18] Blignaut M, Harries S, Lochner A, Huisamen B. Ataxia telangiectasia mutated protein kinase: a potential master puppeteer of oxidative stress-induced metabolic recycling. Oxidative Med Cellul Longev. 2021;2021. 10.1155/2021/8850708 PMC8032526 33868575

[19] Choy KR, Watters DJ (2018). Neurodegeneration in ataxia-telangiectasia: multiple roles of ATM kinase in cellular homeostasis. Dev Dyn.

[20] Pizzamiglio L, Focchi E, Antonucci F (2020). ATM protein kinase: old and new implications in neuronal pathways and brain circuitry. Cells.

[21] Shiloh Y (2020). The cerebellar degeneration in ataxia-telangiectasia: a case for genome instability. DNA Repair.

[22] Brown KD, Ziv Y, Sadanandan SN, Chessa L, Collins FS, Shiloh Y, Tagle DA. The ataxia-telangiectasia gene product, a constitutively expressed nuclear protein that is not up-regulated following genome damage. Proc Natl Acad Sci U S A. 1997;94(5):1840–5. 10.1073/pnas.94.5.1840 PMC20004 9050866

[23] Gately DP, Hittle JC, Chan GKT, Yen TJ. Characterization of ATM expression, localization, and associated DNA-dependent protein kinase activity. Mol Biol Cell. 1998;9(9):2361–74. 10.1091/mbc.9.9.2361 PMC25502 9725899

[24] Boehrs JK, He J, Halaby M, Yang D (2007). Constitutive expression and cytoplasmic compartmentalization of ATM protein in differentiated human neuron-like SH-SY5Y cells. J Neurochem.

[25] Oka A, Takashima S. Expression of the ataxia-telangiectasia gene (ATM) product in human cerebellar neurons during development. Neurosci Lett. 1998;252(3):195–8. 10.1016/s0304-3940(98)00576-x 9739994

[26] Khalil HS, Tummala H, Hupp TR, Zhelev N (2012). Pharmacological inhibition of ATM by KU55933 stimulates ATM transcription. Exp Biol Med.

[27] Gueven N, Fukao T, Luff J, Paterson C, Kay G, Kondo N, Lavin MF (2006). Regulation of the Atm promoter in vivo. Genes Chromosom Cancer.

[28] Miller JA, Ding S-L, Sunkin SM, Smith KA, Ng L, Szafer A, Ebbert A, Riley ZL, Royall JJ, Aiona K (2014). Transcriptional landscape of the prenatal human brain. Nature.

[29] Bindea G, Mlecnik B, Hackl H, Charoentong P, Tosolini M, Kirilovsky A, Fridman WH, Pagès F, Trajanoski Z, Galon J (2009). ClueGO: A Cytoscape plug-in to decipher functionally grouped gene ontology and pathway annotation networks. Bioinformatics.

[30] Ábrahám H, Tornóczky T, Kosztolányi G, Seress L (2001). Cell formation in the cortical layers of the developing human cerebellum. Int J Dev Neurosci.

[31] Ambrose M, Gatti RA (2013). Pathogenesis of ataxia-telangiectasia: the next generation of ATM functions. Blood J Am Soc Hematol.

[32] Lee J-H, Mand MR, Kao C-H, Zhou Y, Ryu SW, Richards AL, Coon JJ, Paull TT. ATM directs DNA damage responses and proteostasis via genetically separable pathways. Sci Signal. 2018;11(512):eaan5598. 10.1126/scisignal.aan5598 PMC5898228 29317520

[33] Maciejczyk M, Heropolitanska-Pliszka E, Pietrucha B, Sawicka-Powierza J, Bernatowska E, Wolska-Kusnierz B, Pac M, Car H, Zalewska A, Mikoluc B (2019). Antioxidant defense, redox homeostasis, and oxidative damage in children with ataxia telangiectasia and nijmegen breakage syndrome. Front Immunol.

[34] Maciejczyk M, Mikoluc B, Pietrucha B, Heropolitanska-Pliszka E, Pac M, Motkowski R, Car H (2017). Oxidative stress, mitochondrial abnormalities and antioxidant defense in Ataxia-telangiectasia, Bloom syndrome and Nijmegen breakage syndrome. Redox Biol.

[35] Howarth C, Gleeson P, Attwell D (2012). Updated energy budgets for neural computation in the neocortex and cerebellum. J Cereb Blood Flow Metab.

[36] Chow H-M, Cheng A, Song X, Swerdel MR, Hart RP, Herrup K (2019). ATM is activated by ATP depletion and modulates mitochondrial function through NRF1. J Cell Biol.

[37] Alexander A, Walker CL (2010). Differential localization of ATM is correlated with activation of distinct downstream signaling pathways. Cell Cycle.

[38] Alexander A, Cai S-L, Kim J, Nanez A, Sahin M, MacLean KH, Inoki K, Guan K-L, Shen J, Person MD. ATM signals to TSC2 in the cytoplasm to regulate mTORC1 and autophagy in response to ROS. Proc Natl Acad Sci USA. 2010;107(9):4153–8. 10.1073/pnas.0913860107 PMC2840158 20160076

[39] Lee J-H, Paull TT (2020). Mitochondria at the crossroads of ATM-mediated stress signaling and regulation of reactive oxygen species. Redox Biol.

[40] Bermúdez-Guzmán L, Leal A (2019). DNA repair deficiency in neuropathogenesis: when all roads lead to mitochondria. Trans Neurodegener.

[41] Fang EF, Kassahun H, Croteau DL, Scheibye-Knudsen M, Marosi K, Lu H, Shamanna RA, Kalyanasundaram S, Bollineni RC, Wilson MA (2016). NAD+ replenishment improves lifespan and healthspan in ataxia telangiectasia models via mitophagy and DNA repair. Cell Metab.

[42] Yang B, Dan X, Hou Y, Lee J, Wechter N, Krishnamurthy S, Kimura R, Babbar M, Demarest T, McDevitt R (2021). NAD+ supplementation prevents STING-induced senescence in ataxia telangiectasia by improving mitophagy. Aging Cell.

[43] Ambrose M, Goldstine JV, Gatti RA (2007). Intrinsic mitochondrial dysfunction in ATM-deficient lymphoblastoid cells. Hum Mol Genet.

[44] Yeo AJ, Chong KL, Gatei M, Zou D, Stewart R, Withey S, Wolvetang E, Parton RG, Brown AD, Kastan MB (2021). Impaired endoplasmic reticulum-mitochondrial signaling in ataxia-telangiectasia. Iscience.

[45] Eaton JS, Lin ZP, Sartorelli AC, Bonawitz ND, Shadel GS (2007). Ataxia-telangiectasia mutated kinase regulates ribonucleotide reductase and mitochondrial homeostasis. J Clin Investig.

[46] Valentin-Vega YA, Kastan MB (2012). A new role for ATM: regulating mitochondrial function and mitophagy. Autophagy.

[47] Cirotti C, Rizza S, Giglio P, Poerio N, Allega MF, Claps G, Pecorari C, Lee J, Benassi B, Barilà D (2021). Redox activation of ATM enhances GSNOR translation to sustain mitophagy and tolerance to oxidative stress. EMBO Rep.

[48] Herrup K, Li J, Chen J (2013). The role of ATM and DNA damage in neurons: upstream and downstream connections. DNA Repair.

[49] Li J, Chen J, Ricupero CL, Hart RP, Schwartz MS, Kusnecov A, Herrup K (2012). Nuclear accumulation of HDAC4 in ATM deficiency promotes neurodegeneration in ataxia telangiectasia. Nat Med.

[50] Li J, Hart RP, Mallimo EM, Swerdel MR, Kusnecov AW, Herrup K (2013). EZH2-mediated H3K27 trimethylation mediates neurodegeneration in ataxia-telangiectasia. Nat Neurosci.

[51] Stagni V, Ferri A, Cirotti C, Barilà D (2021). ATM kinase-dependent regulation of autophagy: a key player in senescence?. Front Cell Dev Biol.

[52] Yang Y, Herrup K (2005). Loss of neuronal cell cycle control in ataxia-telangiectasia: a unified disease mechanism. J Neurosci.

[53] Li J, Chen J, Vinters HV, Gatti RA, Herrup K (2011). Stable brain ATM message and residual kinase-active ATM protein in ataxia-telangiectasia. J Neurosci.

[54] Zhao J, Zhang L, Lu A, Han Y, Colangelo D, Bukata C, Scibetta A, Yousefzadeh MJ, Li X, Gurkar AU (2020). ATM is a key driver of NF-κB-dependent DNA-damage-induced senescence, stem cell dysfunction and aging. Aging (Albany NY).

[55] Chen H, Ruiz PD, McKimpson WM, Novikov L, Kitsis RN, Gamble MJ (2015). MacroH2A1 and ATM play opposing roles in paracrine senescence and the senescence-associated secretory phenotype. Mol Cell.

[56] Kang C, Xu Q, Martin TD, Li MZ, Demaria M, Aron L, Lu T, Yankner BA, Campisi J, Elledge SJ. The DNA damage response induces inflammation and senescence by inhibiting autophagy of GATA4. Science. 2015; 349(6255). 10.1126/science.aaa5612 PMC4942138 26404840

[57] Shiloh Y, Lederman HM (2017). Ataxia-telangiectasia (AT): an emerging dimension of premature ageing. Ageing Res Rev.

[58] Zhang X, Liu P, Zheng X, Wang J, Peng Q, Li Z, Wei L, Liu C, Wu Y, Wen Y, Yan Q, Ma J (2021). N6-methyladenosine regulates atm expression and downstream signaling. HortScience.

[59] Bourseguin J, Cheng W, Talbot E, Hardy L, Lai J, Jeffries AM, Lodato MA, Lee EA, Khoronenkova SV (2022). Persistent DNA damage associated with ATM kinase deficiency promotes microglial dysfunction. Nucleic Acids Res.

[60] Petersen AJ, Rimkus SA, Wassarman DA. ATM kinase inhibition in glial cells activates the innate immune response and causes neurodegeneration in Drosophila. Proc Natl Acad Sci USA. 2012;109(11):E656–64. 10.1073/pnas.1110470109 PMC3306708 22355133

[61] Song X, Ma F, Herrup K (2021). Accumulation of cytoplasmic DNA due to ATM deficiency activates the microglial viral response system with neurotoxic consequences. J Neurosci.

