# Supplementary figures and images for: Disproportionate Expression of ATM in Cerebellar Cortex During Human Neurodevelopment
Source: Cerebellum. 2023 Apr 29;23(2):502–11. doi: 10.1007/s12311-023-01560-2 (PMC10951037; doi:10.1007/s12311-023-01560-2)

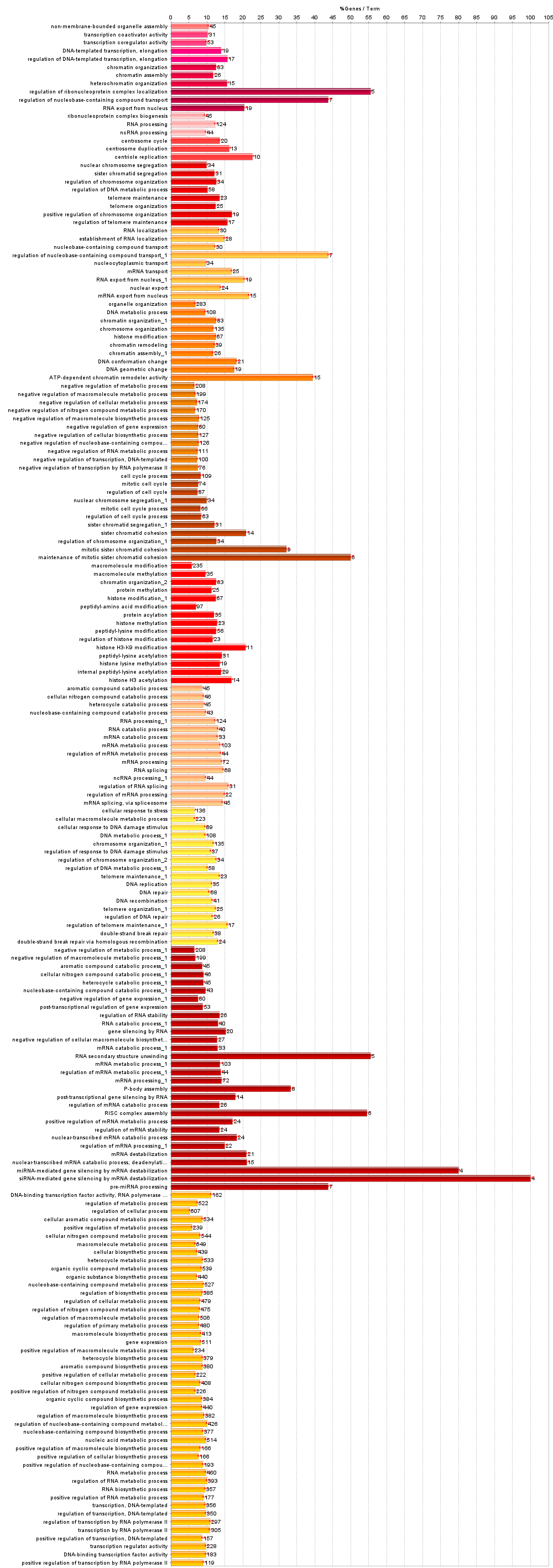

Supplement: Supplementary file 7 — Supplementary file7 (PNG 351 KB) [file 12311_2023_1560_MOESM7_ESM.png]

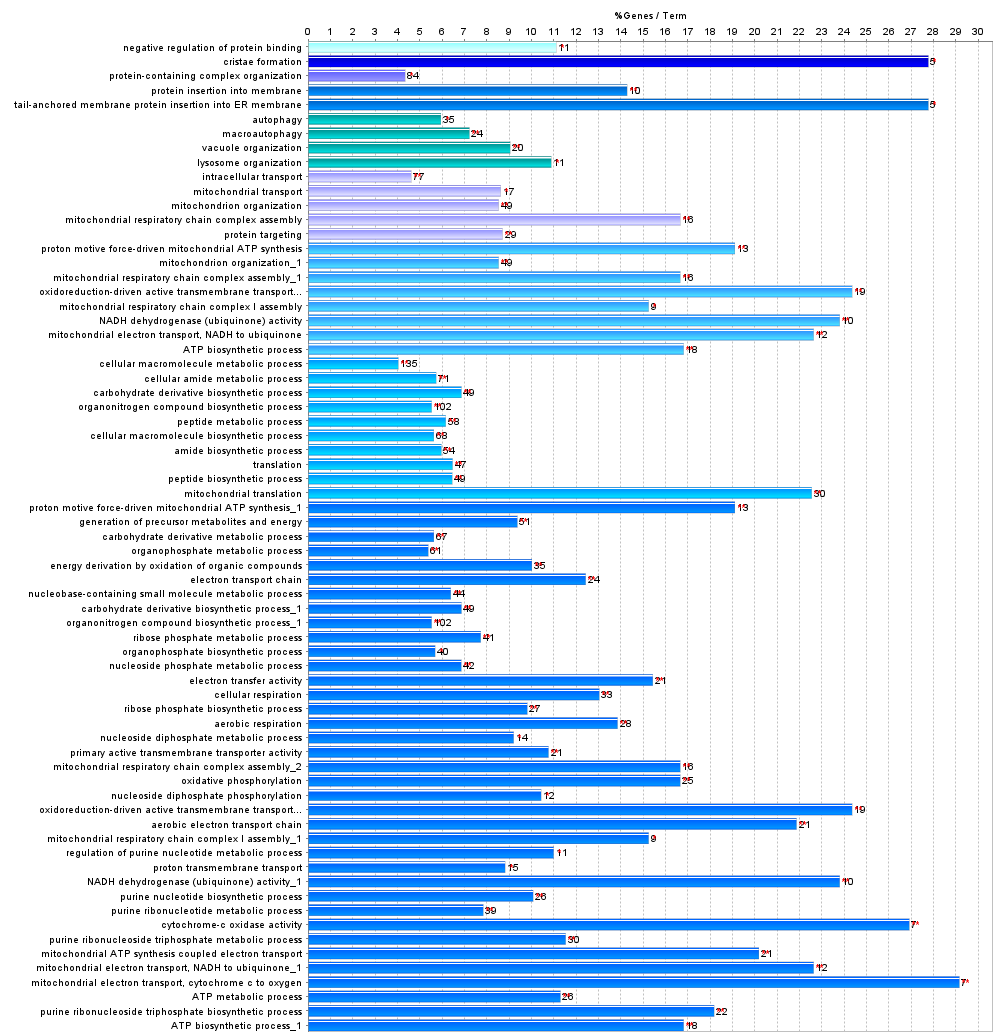

Supplement: Supplementary file 8 — Supplementary file8 (PNG 125 KB) [file 12311_2023_1560_MOESM8_ESM.png]
